# δ-Tocotrienol, a natural form of vitamin E, inhibits pancreatic cancer stem-like cells and prevents pancreatic cancer metastasis

**DOI:** 10.18632/oncotarget.15767

**Published:** 2017-02-28

**Authors:** Kazim Husain, Barbara A Centeno, Domenico Coppola, Jose Trevino, Said M Sebti, Mokenge P Malafa

**Affiliations:** ^1^ Departments of Gastrointestinal Oncology, Tampa, FL, USA; ^2^ Departments of Pathology, Tampa, FL, USA; ^3^ Departments of Drug Discovery, H. Lee Moffitt Cancer Center and Research Institute, Tampa, FL, USA; ^4^ Department of Surgery, University of Florida, Gainesville, FL, USA

**Keywords:** VEDT, pancreatic cancer, metastasis, invasion, EMT

## Abstract

The growth, metastasis, and chemotherapy resistance of pancreatic ductal adenocarcinoma (PDAC) is characterized by the activation and growth of tumor-initiating cells in distant organs that have stem-like properties. Thus, inhibiting growth of these cells may prevent PDAC growth and metastases. We have demonstrated that δ-tocotrienol, a natural form of vitamin E (VEDT), is bioactive against cancer, delays progression, and prevents metastases in transgenic mouse models of PDAC. In this report, we provide the first evidence that VEDT selectively inhibits PDAC stem-like cells. VEDT inhibited the viability, survival, self-renewal, and expression of Oct4 and Sox2 transcription factors in 3 models of PDAC stem-like cells. In addition, VEDT inhibited the migration, invasion, and several biomarkers of epithelial-to-mesenchymal transition and angiogenesis in PDAC cells and tumors. These processes are critical for tumor metastases. Furthermore, in the L3.6pl orthotopic model of PDAC metastases, VEDT significantly inhibited growth and metastases of these cells. Finally, in an orthotopic xenograft model of human PDAC stem-like cells, we showed that VEDT significantly retarded the growth and metastases of gemcitabine-resistant PDAC human stem-like cells. Because VEDT has been shown to be safe and to reach bioactive levels in humans, this work supports investigating VEDT for chemoprevention of PDAC metastases.

## INTRODUCTION

Pancreatic ductal adenocarcinoma (PDAC) is the most lethal cancer, with a dismal 5-year survival rate of 8% [1]. Approximately 25% of patients with PDAC have localized disease that is amenable to a curative approach with surgical resection combined with adjuvant chemotherapy. However, the prognosis of these patients remains poor, with a 5-year overall survival rate of only 28.9% [2, 3]. Therefore, there remains an unmet need to improve outcomes at preventing PDAC relapse.

Recent studies have implicated pancreatic cancer stem-like cells (CSCs) that disseminate from the primary tumor (metastasis-initiating cells) as a critical component of the mechanisms underlying PDAC relapse [4–7]. Pancreatic CSCs express the cell surface markers CD44, CD24, and ESA. They make up 0.2% to 0.8% of human PDAC tumors [8]. These CSCs have the capacity to self-renew, to generate differentiated progeny, are highly tumorigenic and metastatic, and are resistant to chemotherapies used to prevent PDAC relapse [9]. Therefore, a new strategy targeting these CSCs may lead to prevention or delay in PDAC relapse.

Recently, bioactive food components have been shown to alter key oncogenic pathways in pancreatic cancer. Among all 8 natural vitamin E compounds, we have shown that δ-tocotrienol, a natural form of vitamin E (VEDT), is the most potent vitamin E against pancreatic cancer cells [10]. Tocotrienols are a unique family of four natural vitamin E compounds (α-, β-, δ-, γ-tocotrienols), which are found in fruits, vegetables, cereal grains, and essential oils [11] and have distinct biologic activity from tocopherols (α-, β-, δ-, γ) [12]. Preclinical studies have shown that, in contrast to tocopherols, tocotrienols have unique bioactive properties against cancer cells [10, 11, 13–15]. However, the association between pancreatic CSCs and VEDT activity has not been reported.

We have shown that VEDT delays pancreatic cancer progression and improves the survival of genetically engineered mouse models of pancreatic oncogenesis (*LSL-KRAS^G12D^/PDX-1-Cre* and *LSL-Kras^G12D/+^; LSL-Trp53^R172H/+^; Pdx-1-Cre* [KPC]) [16, 17]. We further observed that VEDT significantly inhibited metastases and biomarkers of metastases in the KPC model. Therefore, we postulated that VEDT might inhibit pancreatic CSCs and prevent metastases. In this report, we show that treatment with VEDT markedly inhibited the viability, survival, and self-renewal of pancreatic CSCs. Furthermore, VEDT significantly inhibited biomarkers of processes that underlie the mechanisms of metastasis such as migration, invasion, epithelial-to-mesenchymal transition (EMT), and angiogenesis. Finally, consistent with its *in vitro* activity, VEDT significantly inhibited the growth and metastasis of human PDAC cells and pancreatic CSCs in orthotopic xenograft mouse models. These data provide a rationale for the clinical investigation of VEDT to prevent PDAC relapse.

## RESULTS

### VEDT suppresses growth, self-renewal, and pluripotency factors and induces apoptosis in pancreatic cancer stem cells

**Figure 1:**
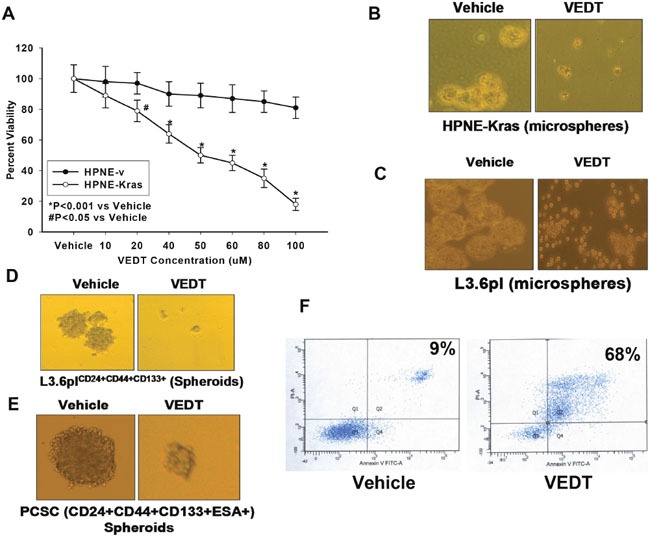
**A**. Vitamin E delta-tocotrienol (VEDT) significantly inhibited proliferation of Kras-transformed human pancreatic normal epithelial cells (HPNE-Kras) in a concentration-dependent manner compared with vehicle (*P<0.001 and #P<0.05) without affecting the immortalized HPNE. **B**. VEDT (50 μM) inhibited the microsphere formation of HPNE-Kras when grown in 3D ultra-low non-adherent culture plates containing specified stem cell medium compared with vehicle. **C**. VEDT (50 μM) inhibited microsphere formation of highly metastatic human pancreatic cancer cells L3.6pl cells when grown in 3D ultra-low non-adherent culture plates containing specified stem cell medium compared with vehicle. **D**. VEDT (50 μM) inhibited self-renewal (spheroid formation) of stem cells L3.6pl CD24+CD44+CD133+ isolated from L3.6pl cells when grown in 3D ultra-low non-adherent culture plates containing specified stem cell medium compared with vehicle. **E**. VEDT (50 μM) inhibited self-renewal (spheroid formation) of human pancreatic CSC (PCSC) CD24+CD44+CD133+ ESA+ when grown in 3D ultra-low non-adherent culture plates containing specified stem cell medium compared with vehicle. **F**. VEDT (10 μM for 5 days) induced apoptosis (Annexin V/PI assay) in human CSCs CD24+CD44+CD133+ ESA+ compared with vehicle control. Results are means ± SE (bars; n = 3). All statistical analyses were performed using ANOVA with Duncan.

One of the features of VEDT bioactivity is its selective inhibition of transformed cells. Using human pancreatic normal epithelial cells (HPNE) and Kras-transformed HPNE cells (HPNE-Kras) in an MTT assay, we confirmed that VEDT significantly inhibited the proliferation of HPNE-Kras cells in a concentration-dependent manner without affecting the HPNE cells (Figure 1A). To test VEDT activity in transformed pancreatic epithelial cells with stem-like features, HPNE-Kras cells were grown in three-dimensional ultra-low non-adherent culture plates containing specific stem cell medium to select for HPNE-Kras stem cells. These cells formed pancreatic microspheres, a characteristic of stem cell self-renewal (Figure 1B). VEDT (50 μM) inhibited the microsphere formation of Kras-transformed HPNE cells compared with vehicle (Figure 1B). Similarly, when the highly metastatic pancreatic cancer cells L3.6pl were grown in stem cell medium, they also formed pancreatic microspheres (Figure 1C). VEDT (50 μM) also inhibited microsphere formation of L3.6pl cells compared with vehicle (Figure 1C). We further investigated the effects of VEDT on self-renewal (spheroid formation) of pancreatic CSCs L3.6pl CD24+CD44+CD133+ isolated from the pancreatic cancer cell line L3.6pl using flow cytometry and the commercially available patient-derived human (parental) pancreatic CSCs CD24+CD44+CD133+ ESA+ grown in three-dimensional ultra-low non-adherent culture plates containing stem cell medium (Figure 1D and 1E). VEDT (50 μM) inhibited the self-renewal (spheroid formation) of both human pancreatic CSCs compared with vehicle (Figure 1D and 1E). In addition, we investigated the effects of VEDT on apoptosis in pancreatic CSCs. VEDT (10 μM) exposure for 5 days induced significant apoptosis (68%) compared with vehicle (9%) in human pancreatic CSCs CD24+CD44+CD133+ ESA+ (Figure 1F).

**Figure 2:**
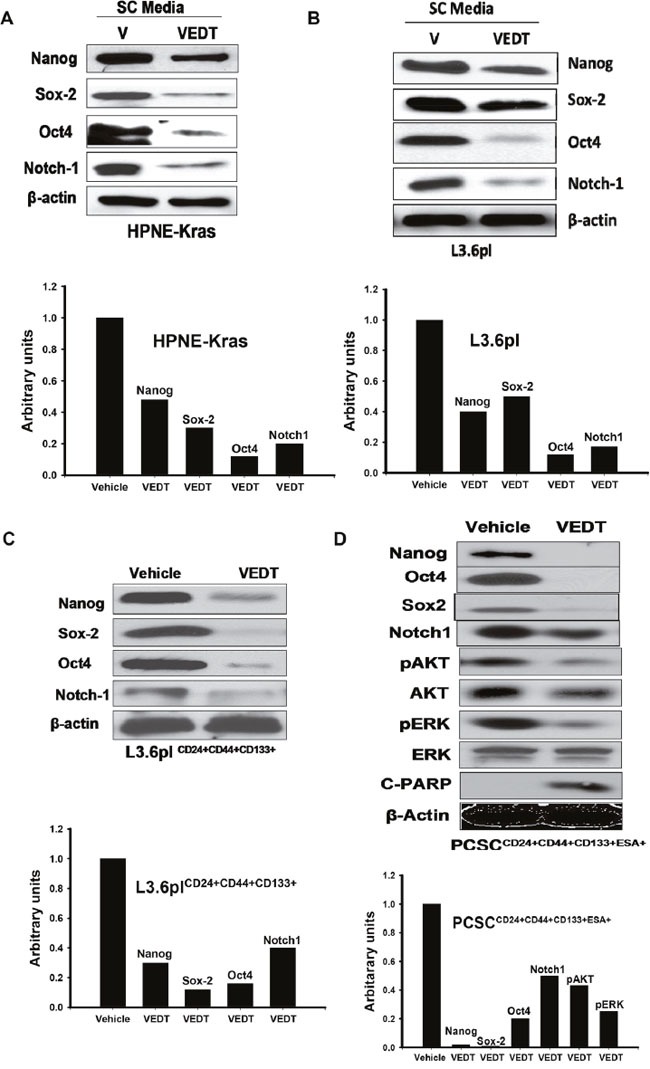
**A**. Western blot analysis and densitometric analyses of HPNE-Kras grown in stem cell (SC) medium and treated with VEDT or vehicle (V) for 24 hours on stem cell pluripotency transcription factors. VEDT (50 μM) inhibited the expression of transcription factors Nanog, Oct4, and Sox2 compared with vehicle. VEDT also inhibited Notch1 receptor expression compared with vehicle control. **B**. Western blot analysis and densitometric analyses of highly metastatic human pancreatic cancer cells (L3.6pl) grown in SC medium and treated with VEDT or vehicle for 24 hour on stem cell pluripotency transcription factors. VEDT (50 μM) inhibited expression of Nanog, Oct4, and Sox2 compared with vehicle. VEDT also inhibited Notch1 receptor expression compared with vehicle control. **C**. Western blot analysis and densitometric analyses of pancreatic CSCs L3.6pl CD24+CD44+CD133+ isolated from L3.6pl cells grown in SC medium and treated with VEDT or vehicle for 24 hours on stem cell pluripotency transcription factors. VEDT (50 μM) inhibited the expression of Nanog, Oct4, and Sox2 compared with vehicle. VEDT also inhibited the Notch1 receptor expression compared with vehicle control. **D**. Western blot analysis and densitometric analyses of human pancreatic CSCs (PCSC CD24+CD44+CD133+ESA+) grown in SC medium and treated with VEDT or vehicle for 24 hours on stem cell pluripotency transcription factors, Kras downstream signaling, and apoptosis. VEDT (50 μM) inhibited the expression of Nanog, Oct4, and Sox2 compared with vehicle. VEDT also inhibited Notch1 receptor expression and pAKT and pERK expression and induced apoptosis (C-PARP) compared with vehicle control. All experiments were performed in triplicate.

As shown in Figure 2D, Western blot data confirmed VEDT-induced apoptosis (C-PARP) compared with vehicle in pancreatic CSCs. To further characterize pancreatic CSCs after VEDT treatment, we assessed the expression of stem cell-associated regulatory proteins. Western blot data presented in Figure 2A–2D show that VEDT further inhibited expression levels of the stem cell transcription factors Nanog, Oct4, and Sox2 compared with vehicle in pancreatic cancer cells grown in stem cell media as well as in pancreatic CSCs. VEDT also inhibited the Notch1 receptor and Kras downstream signaling factors pAKT and pERK in pancreatic CSCs (Figure 2D). Taken together, these results indicate that VEDT suppresses the growth, survival, and self-renewal of pancreatic CSCs and suppresses the expression of stem cell regulatory factors in PDAC stem-like cells.

### VEDT inhibits migration and invasion of pancreatic cancer L3.6pl and MiaPaCa-2 cells

**Figure 3:**
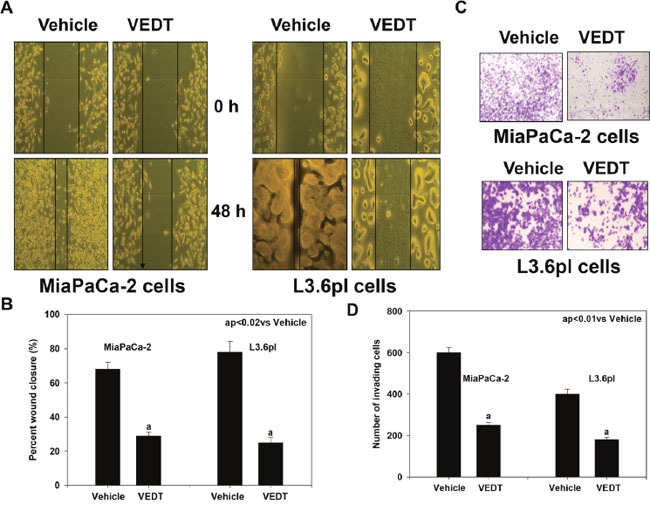
**A**. Effect of VEDT on cell migration of human pancreatic cancer cell lines MiaPaCa-2 and L3.6pl after 48 hours (wound healing assay or scratch test). **B**. VEDT (50 μM) significantly inhibited cell migration in MiaPaCa-2 and L3.6pl cells compared with vehicle control (^a^p<0.02). **C**. Effect of VEDT on invasion of MiaPaCa-2 and L3.6pl cells (Matrigel invasion chamber test). **D**. VEDT (50 μM) significantly inhibited cell invasion in MiaPaCa-2 and L3.6pl cells compared with vehicle control (^a^P<0.01). Results are means ± SE (bars; n = 3). All statistical analyses were performed using ANOVA with Duncan.

To establish a metastatic foci, cancer cells need to migrate from the primary tumor to invade tissues. We next investigated whether VEDT could also inhibit the metastatic processes of cell migration and invasion in pancreatic cancer L3.6pl and Mia PaCa-2 cells. Using the wound healing assay, we found that VEDT significantly (P<0.01) suppressed tumor cell mobility in both pancreatic cancer cells compared with their corresponding vehicle controls (Figure 3A and 3B). Similarly, invasion assay with Matrigel demonstrated that VEDT significantly (P<0.01) decreased the invasive capacity of pancreatic cancer L3.6pl and Mia PaCa-2 cells compared with vehicle (Figure 3C and 3D). Taken together, these results suggest that VEDT can suppress pancreatic cancer cell migration and invasion *in vitro*, thereby indicating the capacity to inhibit metastatic spread.

### VEDT inhibits epithelial-to-mesenchymal transition in pancreatic cancer cells and tumors

**Figure 4:**
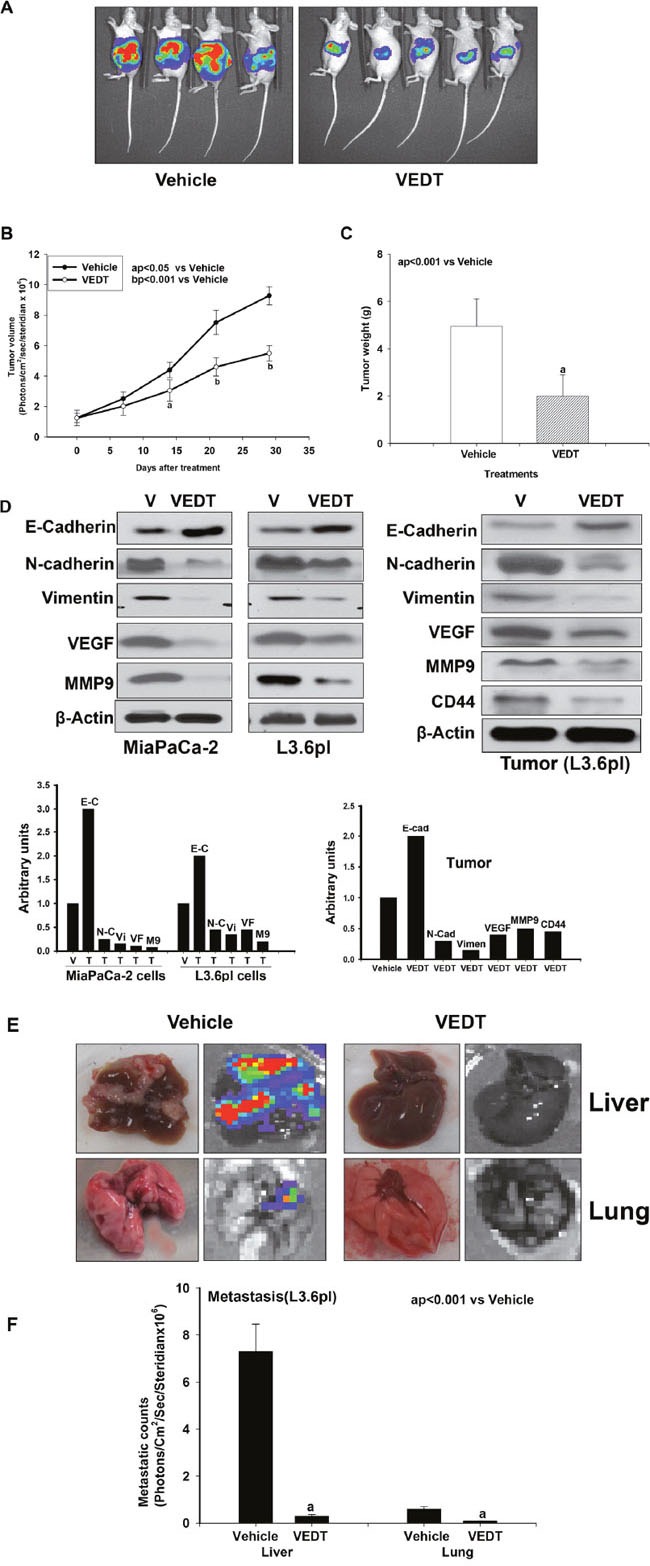
**A**. VEDT (200 mg/kg, PO twice a day) inhibited pancreatic (L3.6pl) tumor growth compared with vehicle in mice. **B**. VEDT significantly decreased pancreatic tumor volume 2 weeks after treatment compared with vehicle (^a^P<0.05) and more significantly (^b^P<0.001) after 3-4 weeks of treatment compared with vehicle control. **C**. VEDT significantly decreased pancreatic tumor weight 4 weeks after treatment compared with vehicle (^a^P<0.001). **D**. Western blot analysis and densitometric analyses of epithelial-to-mesenchymal transition (EMT) markers, metastasis, and angiogenesis in MiaPaCa-2 and L3.6pl cells and in tumor tissues of mice. VEDT (50 μM) induced epithelial cell marker (E-cadherin) and inhibited mesenchymal cell marker (N-cadherin and vimentin) in MiaPaCa-2 and L3.6pl cells and in tumor tissues compared with vehicle. VEDT (50 μM) inhibited metastasis (MMP9) and angiogenesis (VEGF) in MiaPaCa-2 and L3.6pl cells as well as cell surface marker of CSCs (CD44) in tumor tissues compared with vehicle. Data are from 3 independent experiments. **E**. VEDT inhibited liver and lung metastasis in mice. **F**. VEDT significantly inhibited both liver and lung metastasis (^a^P<0.001) compared with vehicle control. Results are means ± SE (bars; n = 3-5). All statistical analyses were performed using ANOVA with Duncan.

Epithelial-to-mesenchymal transition (EMT) is a process whereby epithelial cells lose their cell-cell adhesion and gain migratory and invasive properties. EMT enables cancer cell invasion, which is the initial step in metastases. Furthermore, cells that undergo EMT gain stem cell-like properties thought to give rise to CSCs. To assess the effects of VEDT on EMT *in vitro*, we used Western blot analyses to examine the expression of epithelial (E-cadherin) and mesenchymal markers (N-cadherin and vimentin) in L3.6pl and Mia PaCa-2 cells exposed to VEDT *in vitro* and *in vivo*. VEDT significantly (P<0.05) increased E-cadherin expression in L3.6pl and MiaPaCa-2 cells as well as in tumor tissue compared with vehicle control (Figure 4D). In contrast, there were significant decreases in the expression levels of N-cadherin and vimentin in L3.6pl and Mia PaCa-2 cells and in tumor tissue compared with vehicle control (Figure 4D). These data demonstrate that VEDT is an inhibitor of EMT in pancreatic cancer cells.

### VEDT inhibits biomarkers of tumor angiogenesis and metastasis and induces apoptosis in pancreatic cancer cells and CSCs and in pancreatic tumors

**Figure 5:**
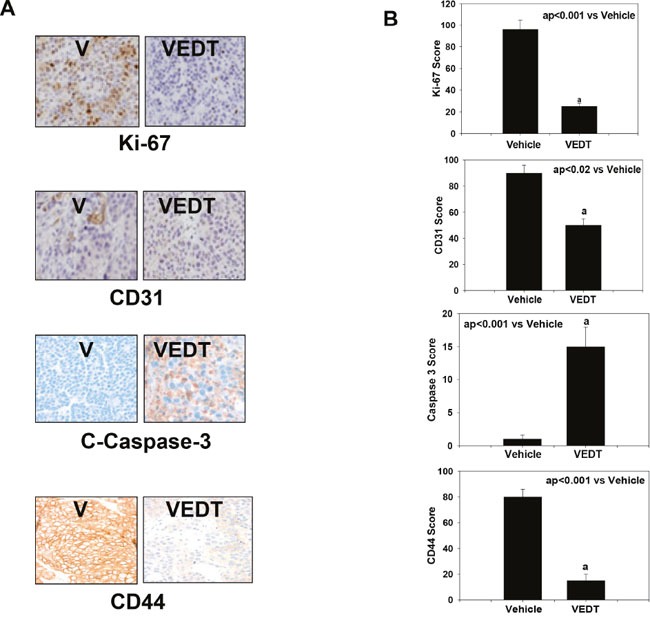
**A**. Effect of VEDT on immunostaining of pancreatic tumor proliferation (Ki-67), angiogenesis (CD31), cell surface marker of CSCs (CD44), and apoptosis (cleaved (C) caspase 3) in mice. **B**. VEDT significantly inhibited proliferation index of the pancreatic tumor (^a^P<0.001), angiogenesis (^a^P<0.02), and CSCs (^a^P<0.001) and induced apoptosis (^a^P<0.001) compared with vehicle control in mice. Results are mean and SE (bars; n = 5). All statistical analyses were performed using ANOVA with Duncan.

To investigate the effects of VEDT on biomarkers of angiogenesis, we used Western blot and immunohistochemistry analyses to examine the expression of VEGF and CD31 respectively *in vitro* using L3.6pl and Mia PaCa-2 cells as well as CSCs *in vivo* using tumors. Matrix metallopeptidase 9 (MMP9) is a critical enzyme that is involved in the degradation of extracellular matrix, thereby facilitating angiogenesis, invasion, and metastasis. Therefore, we evaluated the effect of VEDT on MMP9 in pancreatic cancer cells and tumor. VEDT depleted VEGF and MMP9 expression in pancreatic cancer L3.6pl and MiaPaCa-2 cells (Figure 4D) and in pancreatic tumor tissue (CD31) compared with vehicle control (Figure 5). In the tumor tissues, the cell surface marker of CSCs (CD44) was significantly depleted after VEDT treatment compared with vehicle as analyzed by both immunostaining (Figure 5A and 5B) and Western blot (Figure 4D) analyses.

The intensities of CD31 and CD44 immunostaining were significantly (P<0.02 and P<0.001) decreased in tumor tissue compared with vehicle control (Figure 5A and 5B). Moreover, as previously observed by us and others, VEDT significantly (P<0.001) induced apoptosis (cleaved caspase 3 immunostaining) in pancreatic tumors compared with vehicle treatment (Figure 5A and 5B).

### VEDT inhibits pancreatic tumor growth and metastasis in mice

To further determine the effects of VEDT on pancreatic tumor growth and metastasis *in vivo*, L3.6pl cells expressing luciferase were inoculated orthotopically into the pancreas of nude mice, and the animals were randomized to treatment groups and closely monitored for tumor growth for 4 weeks. The results show that tumor volume, weight and proliferation index (Ki-67) staining were significantly decreased in VEDT treated compared with vehicle treated tumors (Figure 4A–4C and Figure 5A and 5B). Furthermore, we examined the effects of VEDT on liver and lung metastasis *in vivo*. As shown in Figure 4E and 4F, the amounts of liver and lung metastasis nodules were dramatically decreased in the VEDT group compared with the vehicle group, as measured by luciferase activity. In addition to the depletion of MMP9 in cells and in the tumor tissue of mice treated with VEDT compared with vehicle shown in Figures 4 and 5, together, these results indicate that VEDT can suppress the tumorigenesis and metastasis of pancreatic cancer cells *in vitro* and *in vivo*.

**Figure 6:**
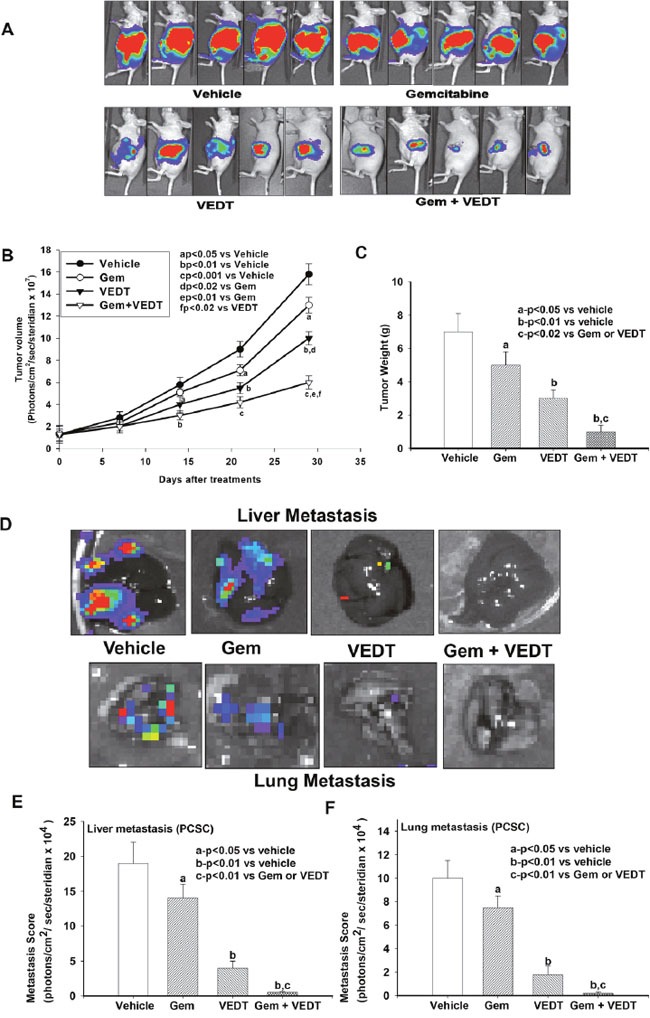
**A**. Effect of gemcitabine (100 mg/kg, IP twice a week) and VEDT (200 mg/kg, PO twice a day) alone and in combination for 4 weeks on inhibition of human pancreatic CSC tumor growth compared with vehicle in mice. **B**. Gemcitabine significantly decreased pancreatic tumor volume 3 to 4 weeks after treatment (^a^P<0.05) compared with vehicle. VEDT significantly decreased pancreatic tumor volume 2 weeks after treatment compared with vehicle (^a^P<0.05) and more significantly (^b^P<0.01) after 3-4 weeks of treatment compared with vehicle control. The 2 drugs combined decreased the tumor volume significantly compared to vehicle (^b^P<0.01) after 2 weeks of treatment and more significantly (^c^P<0.001) after 3-4 weeks of treatment compared to vehicle. The combination was more effective in reducing the tumor volume compared with either drug alone **C**. Gemcitabine significantly decreased pancreatic tumor weight 4 weeks after treatment compared with vehicle (^a^P<0.05). VEDT significantly decreased pancreatic tumor weight 4 weeks after treatment compared with vehicle (^b^P<0.01) or compared with gemcitabine (^c^P<0.02). The combination of the drugs was more effective in reducing the tumor weight than either drug alone. **D**. Effect of gemcitabine and VEDT alone and in combination on liver and lung metastasis in mice. **E**. Gemcitabine slightly but significantly reduced liver metastasis compared with vehicle (^a^P<0.05). VEDT significantly more inhibited liver metastasis (^b^P<0.01) compared with vehicle control. The combination of the 2 drugs completely and significantly abolished liver metastasis in mice (^c^P<0.01). **F**. Gemcitabine slightly but significantly reduced lung metastasis compared with vehicle (^a^P<0.05). VEDT significantly more inhibited lung metastasis (^b^P<0.01) compared to vehicle. However, the combination of the 2 drugs completely and significantly abolished lung metastasis in mice (^c^P<0.01). Results are means ± SE (bars; n = 5). All statistical analyses were performed using ANOVA with Duncan.

We further investigated the effects of gemcitabine, the present standard of care, and VEDT alone and in combination on pancreatic tumor growth and metastasis in mice orthotopically injected with the pancreatic CSCs expressing luciferase into the pancreas. As shown in Figure 6A–6C, gemcitabine slightly decreased pancreatic tumor volume and weight 4 weeks after treatment (P<0.05) compared with vehicle. On the other hand, VEDT more significantly decreased pancreatic tumor volume and weight (P<0.01) after 4 weeks of treatment compared with vehicle. However, the combination of the two drugs resulted in the most significant reduction of tumor volume and weight compared with vehicle (P<0.001) after 4 weeks of treatment. The combination was more effective in reducing the tumor volume and weight compared with either drug alone. As shown in Figure 6D–6F, the liver and lung metastasis scores were slightly decreased with gemcitabine treatment but profoundly decreased in the VEDT group compared with vehicle group (P<0.01). The combination of the two drugs completely and significantly abolished liver and lung metastasis in mice.

## DISCUSSION

Vitamin E delta-tocotrienol (VEDT), a natural vitamin E compound, is receiving increasing attention as a potential chemopreventive and/or chemotherapeutic agent [14, 18, 19]. Several studies *in vitro* and *in vivo* have demonstrated antitumor activity of VEDT [10, 16, 17, 20–24]. Moreover, we have shown in early-phase clinical trials that VEDT is well tolerated and reached bioactive levels in humans [25, 26]. We describe in this study the activity of VEDT on pancreatic cancer cells with stem-like features. We showed VEDT selectively inhibited human pancreatic ductal epithelial cells expressing mutated oncogenic Kras. We validated VEDT antitumor activity in different pancreatic cancer cells and two pancreatic cancer xenograft models. We show that it affects motility, invasion, angiogenesis, and induces caspase activation leading to cell death. The efficacy of VEDT in preventing metastasis was demonstrated *in vivo* in a highly resistant human pancreatic cancer stem cell xenograft model both as monotherapy and in combination with gemcitabine.

Although the survival rate of resected pancreatic cancer has recently improved since the introduction of adding capecitabine to the standard adjuvant regimen of gemcitabine, the 5-year survival rate is still 28.9% [2, 3, 27]. Although combination chemotherapy can be effective in the treatment of pancreatic cancer, increasing the number of chemotherapeutic drugs is associated with increasing toxicity with patients remaining at high-risk of relapse [4, 9]. Current studies suggest that cancer relapse is caused by the regrowth of the surviving inherently chemoresistant CSCs that persisted during adjuvant chemotherapy. The expansion of this chemoresistant cancer cell population coupled with the capacity of these cells to be mobile and invade tissues as well as favorable microenvironment is thought to be responsible for the development of chemoresistant recurrent tumors [5, 7, 9]. Therefore, to decrease the risk of relapse and thereby improve survival, a practical approach is to use novel therapies that can induce cell death and to target the features that promote cancer reoccurrence. Indeed, we demonstrated that VEDT is able to induce cell death in pancreatic cancer cells with stem-like characteristics as well as to inhibit self-renewal, motility, and the invasive capacity of pancreatic cancer cells. Moreover, we showed that VEDT is a potent agent against pancreatic tumor metastasis.

The pancreatic cancer xenograft models used in this study recapitulate the clinical profile observed in patients such as liver metastasis and chemoresistance in primary and metastatic tumors [28, 29]. We demonstrated the efficacy of VEDT given as a monotherapy and as part of combination treatment. We show that it is not antagonistic to the current adjuvant standard therapy with gemcitabine. VEDT delays tumor growth kinetics by inhibiting the proliferation and inducing apoptosis. Furthermore, the microenvironment or metastatic niche appears to be inhibited by VEDT.

Pancreatic cancer metastatic dissemination occurs by hematogenous pathway, and its spread is most often found in liver and lung [30]. Metastasis is a highly organ-specific pathophysiologic activity involving multiple steps and interactions between cancer cells and the host, such as proliferation, angiogenesis, invasion, detachment from the primary tumor, invasion into circulatory system, and extravasation into liver and lung and further proliferation and angiogenesis [28, 29, 31]. Furthermore, EMT is a program in which senile epithelial cells are transformed to motile mesenchymal cells [32]. Therefore, induction of EMT can lead to invasion, intravasation, dissemination, and colonization of tumor cells in the liver and lung [30, 33]. Molecular analysis has demonstrated the effects of VEDT on EMT and Kras oncogenic pathways and stem cell transcription factors. VEDT treatment inhibited the EMT induction by enhancing the expression of epithelial marker E-cadherin and decreasing the expression of mesenchymal markers N-cadherin and vimentin in pancreatic cancer cells and in tumor tissues. EMT also plays an important role during metastatic tumor progression through enhanced angiogenesis [34]. Induction of angiogenesis via the sprouting of new vessels from existing ones is considered to be the exclusive method of tumor vascularization [35]. Our earlier study showed that VEDT inhibited the angiogenic factor VEGF protein expression and endothelial factor CD31 immunostaining in the tumor blood vessels of genetic mouse model of pancreatic cancer [16]. Furthermore the down-regulation of E-cadherin and increased activity of matrix metalloproteinase 9 (MMP9) has been reported in metastatic tumors [36]. Our data show that VEDT reversed the EMT and depleted MMP9 expression in the pancreatic tumors.

Tumors are composed of diverse types of cells, and CSCs are at the top of the hierarchical pyramid [37–39]. Evidence has demonstrated that CSCs have the capacity for differentiation along tumor and endothelial lineages as well as vascular smooth muscle-like cells [40–42]. Recent studies have implicated pancreatic CSCs that disseminate from the primary tumor (metastasis-initiating cells) as a critical component of the mechanisms underlying PDAC relapse [4–7]. Pancreatic CSCs express the cell surface markers CD44, CD24, and ESA. They make up 0.2% to 0.8% of human PDAC tumors [8]. VEDT also inhibited the self-renewal capacity (spheroid formation), one of the characteristics of CSCs. VEDT inhibited pluripotency transcription factors such as Nanog, Oct4, and Sox-2, Notch-1 receptor, metastasis marker MMP9 as well as Kras downstream signaling factors pAKT and pERK in pancreatic CSCs, indicating Kras signaling in pancreatic cancer stemness. Although several of our previous studies have investigated how VEDT suppresses oncogenic pathways [10, 43, 44], future studies should focus on the molecular mechanisms of VEDT-induced regulation of Kras signaling in relation to pancreatic CSC transcription factors. Taken together, our data indicate that VEDT, in addition to inhibiting the growth and survival of pancreatic tumor cells, targets the pancreatic CSCs, leading to suppression of liver and lung metastasis.

In summary, we found that natural VEDT inhibits pancreatic cancer metastasis. This function is associated with selective inhibition of the growth and survival of transformed pancreatic cancer cells as well as inhibition of migration, invasion, angiogenesis, and EMT of these cells *in vitro* and *in vivo*. Here, we report for the first time that VEDT also inhibits pancreatic CSC growth, self-renewal, and pluripotency. These data provide a strong rationale for further studies of the mechanisms underlying the bioactivity of VEDT against transformed cells and justifies translation to early-phase clinical trials aimed at reducing the risk and progression of pancreatic cancer.

## MATERIALS AND METHODS

### Chemicals

All chemicals and reagents were purchased from Sigma-Aldrich (St. Louis, MO) unless otherwise specified. Tocotrienol was obtained from Davos Life Ltd (Helios, Singapore). L-glutamine, penicillin, streptomycin, and HEPES buffer were purchased from Mediatech (Herndon, VA, USA). Fetal bovine serum (FBS) was purchased from HyClone (Logan, UT, USA). Dulbecco's modified minimal essential medium (DMEM) and phosphate-buffered saline (PBS) were purchased from Invitrogen (Carlsbad, CA, USA). Ethanol (100%) was purchased from Aaper Alcohol and Chemical (Shelbyville, KY, USA). Immortalized human pancreatic normal epithelial cells with empty vector (HPNE-V) and Kras-transformed HPNE cells (HPNE-Kras) were gifts from Dr. Paul Campbell (Moffitt Cancer Center). M3:F medium was purchased from INCELL (San Antonio, TX). Human pancreatic cancer cell line MiaPaCa-2 and DMEM F12 medium (M36115-42PS) were obtained from ATCC (Manassas, VA). The human highly metastatic pancreatic cancer L3.6pl cell line expressing luciferase was gift from Dr. Jose Trevino (University of Florida, Gainesville, FL, USA). Pancreatic CSCs and growth medium were purchased from Celprogen Inc. (Torrance, CA, USA).

### Cell culture and growth

Immortalized human pancreatic normal epithelial cells with empty vector (HPNE-V) and Kras-transformed HPNE cells (HPNE-Kras) were cultured in a mixture of DMEM and M3:F media at a ratio of 3 parts DMEM and 1 part M3:F media and supplemented with 5% FBS. Human pancreatic cancer cells (MiaPaCa-2 and L3.6pl) and human (parental) pancreatic CSCs were grown in DMEM media supplemented with 10% FBS, 2 mM L-glutamine, penicillin (50 IU/ml), and streptomycin (50 mg/ml) and human pancreatic CSC complete growth medium. The cells were cultured at 37°C in a humidified atmosphere of 5% CO_2_ and 95% O_2_.

### Cell proliferation MTT assay

Cells were seeded in 96-well plates at a density of 3000 cells per well and allowed to attach overnight. Cells were then incubated for 72 hours with various concentrations of VEDT (10^−5^ to 10^−4^ M) or ethanol (<5%) vehicle as control. Media were aspirated and replaced with 20 μL of 1 mg/mL MTT and incubated for 2-4 hours at 37°C in a humidified atmosphere of 5% CO_2_. Media were aspirated and 200 μL of DMSO added to each well and incubated for 5 minutes with shaking, and absorbance was read at 540 nm.

### Cell migration and invasion assay

Cell migration was performed by scratch test or wound healing assay. MiaPaCa-2 and L3.6pl cells were seeded in six-well plates and cultured to 100% confluence. Wounds were generated in the cell monolayer using a small plastic pipette tip. The cells were then rinsed with PBS and cultured for another 48 hours. The spread of wound closure was observed and photographed under light microscope. For invasion assays, 1×10^5^ cells (MiaPaCa-2 and L3.6pl) in serum-free media were added into the upper chamber of an insert precoated with Matrigel (BD Bioscience). The lower chamber was filled with DMEM with 10% FBS. After 48 hours of incubation, the cells remaining on the upper surface of the membrane were removed, whereas the cells that had invaded through the membrane were stained with 20% methanol and 0.2% crystal violet, imaged, and counted under light microscope.

### Isolation of human pancreatic CSCs from L3.6pl spheroids by flow cytometry

Human metastatic pancreatic cancer L3.6pl cells were plated in six-well ultra-low attachment plates (Corning Inc., Corning, NY) at a density of 1,000 cells/mL in DMEM supplemented with 1% N2 Supplement (Invitrogen), 2% B27 Supplement (Invitrogen), 20 ng/mL human platelet growth factor (Sigma-Aldrich), 100 ng/mL epidermal growth factor (Invitrogen), and 1% antibiotic-antimycotic (Invitrogen) at 37°C in a humidified atmosphere of 95% air and 5% CO_2_. Spheroids were collected after 7 days and dissociated with Accutase (Innovative Cell Technologies). Fluorescence-activated cell sorting experiments were carried out by flow cytometry using antibody against stem cell markers CD44-APC, CD24-FITC, and CD133-PE.

### Microsphere formation assay

Kras-transformed HPNE cells (HPNE-Kras) and the L3.6pl human pancreatic cancer cells were plated in three-dimensional culture ultra-low non-adherent plates (Corning Inc. Corning, NY, USA) containing stem cell-specific DMEM/F12 medium supplemented with 2% B27 supplement, 1% N2 supplement, 10 ng/ml bFGF, 20 ng/ml EGF, and 1% antibiotics and treated with vehicle (5% ethanol) and VEDT (50 μM) incubated at 37°C for 5 days. Numbers of microspheres formed were counted under microscope and photographed.

### Spheroid formation (self-renewal) assay

One thousand human (parental) pancreatic CSCs CD24+CD44+CD133+ESA+ and L3.6pl CD24+CD44+CD133 pancreatic CSCs isolated from L3.6pl pancreatic cancer cells by flow cytometry were plated in 3D culture ultra-low non-adherent plates (Corning Inc.) containing stem cell-specific DMEM/F12 medium supplemented with 2% B27 supplement, 1% N2 supplement, 10 ng/ml bFGF, 20 ng/ml EGF, and 1% antibiotics and treated with vehicle (5% ethanol) and VEDT (50 μM) incubated at 37°C for 5 days. Numbers of spheroids formed were counted under microscope and photographed.

### Apoptosis assay

Human (parental) pancreatic cancer stem cells CD24+CD44+CD133+ESA+ were plated and treated concurrently with vehicle (5% ethanol) or VEDT (10 μM) for 5 consecutive days. Cells were harvested, and 10^5^ cells were transferred to 5-mL tube in PBS (100 μL) then 2 μL of PI and 5 μL of Annexin V-FITC (BD Bioscience) were added and mixed. The tubes were incubated for 15 minutes at room temperature in the dark, and then 400 μL of binding buffer was added and tubes were analyzed for apoptosis by flow cytometry.

### Animals and treatments

Female athymic nude mice (6 weeks old, 20-23 g) were obtained from Charles River (Wilmington, MA, USA) and kept in the institute's animal facility for 1 week for quarantine. In the first experiment, mice (n = 10) were orthotopically implanted with luciferase expressing L3.6pl cells (one million in 50 μL) to the pancreas. After 1 week, mice were randomly divided into 2 treatment groups as follows: *1*) normal controls (100 μL oral gavage of ethanol extracted olive oil (vehicle) twice daily for 4 weeks and *2*) VEDT (200 mg/kg oral gavage daily for 4 weeks). In a second experiment, mice (n = 20) were orthotopically implanted with luciferase expressing human (parental) pancreatic CSCs CD24+CD44+CD133+ESA+ (1 × 10^5^ cells in 50 μl) to the pancreas. After 1 week, mice were randomly divided into 4 treatment groups as follows: *1*) normal controls (100 μL oral gavage of ethanol extracted olive oil (vehicle) twice daily for 4 weeks, *2*) gemcitabine (100 mg/kg, IP twice a week for 4 weeks), *3*) VEDT (200 mg/kg oral gavage twice a day for 4 weeks), and *4*) gemcitabine plus VEDT for 4 weeks. The tumor volume was measured once a week using IVIS 200 (Xenogen). After 4 weeks of treatment, the animals were killed and tumor weight, liver, and lung metastasis were recorded and scored. Half of the tumor tissues were immediately immersed in liquid nitrogen and stored at -80°C for biochemical analyses and another half were fixed in buffered formalin for histological analysis. The care and use of the animals reported in this study were approved by Institutional Laboratory Animal Care and Use Committee (IACUC) and as per the guidelines of the National Institute of Health (NIH).

### Cell and tissue protein extraction and protein determination

Cells and tumor tissues were washed thrice in cold PBS (pH 7) and then lysed in protein extraction reagent RIPA buffer (Thermo Scientific, Rockford, IL) containing EDTA and protease inhibitor cocktail. Protein concentration was determined using BCA reagents (Pierce, Rockford, IL) according to the manufacturer's instructions.

### Western blot analysis

Extracted proteins from cells and tumor tissues (40 μg) were resolved on 12.5% SDS polyacrylamide gel (SDS-PAGE) running gel and a 5% stacking gel. Proteins were then electrotransferred onto nitrocellulose membranes. After blocking in 5% nonfat powdered milk for 1 hour, the membranes were washed and then treated with antibodies to E-cadherin, N-cadherin, vimentin, VEGF, MMP9, Nanog, Oct4, Sox-2, CD44, Notch-1, cleaved PARP, pAKT, AKT, pERK, ERK and β-actin (1:1000) for overnight at 4°C (Santa Cruz Biotechnology, Santa Cruz, CA; Cell Signaling, Danvers, MA). After they were washed, blots were incubated with horseradish peroxidase-conjugated secondary antibody IgG (1:5000) for 1 hour at room temperature. The washed blot was then treated with SuperSignal West Pico chemiluminescent substrate (Pierce) for positive antibody reaction. Membranes were exposed to X-ray film (KODAK) for visualization and densitometric quantization of protein bands using AlphaEaseFC software (Alpha Innotech).

### Histologic evaluation

Formalin-fixed, paraffin-embedded tissues were sectioned (4 μm) and stained with hematoxylin-eosin. Immunohistochemistry was performed using the Ventana Discovery XT automated system (Ventana Medical Systems, Tucson, AZ) per manufacturer's protocol with proprietary reagents. Briefly, slides were deparaffinized on the automated system with EZ Prep solution. Sections were heated for antigen retrieval. For immunohistochemistry, tissue sections were incubated with Ki-67, caspase 3, CD44, and CD31 at 1:4000 dilutions for 60 minutes. Detection was performed using the Ventana OmniMap kit.

### Assessment of immunohistochemical expression

All stained tissues were examined by one independent observer (BAC). Ki-67, CD44, and caspase 3 stained tissues were assessed for expression of the observed areas. Percent expression was recorded for each area and then averaged for each mouse. For CD31, sections were examined at low power to identify cancers and associated hot spots. The vessels per x400 field were counted manually. Single cells and groups expressing CD31 were counted as vessels in addition to groups with lumens. Sections not showing a cancer were assessed for hot spots at low power.

### Statistical analyses

The data are expressed as mean ± SEM. The data were analyzed statistically using unpaired t-tests or one-way analysis of variance (ANOVA) where appropriate. ANOVA was followed by Duncan's multiple range tests using SAS statistical software for comparison between different treatment groups. Statistical significance was set at P< 0.05.
